# Taxonomic Revision of Jingle Shells: Resurrecting and Reclassifying Species of Anomiidae (Bivalvia: Pectinida)

**DOI:** 10.1002/ece3.72372

**Published:** 2025-10-23

**Authors:** Yi‐Tao Lin, Jian‐Wen Qiu

**Affiliations:** ^1^ SANYA Oceanographic Laboratory Sanya China; ^2^ Department of Biology Hong Kong Baptist University Hong Kong SAR China

**Keywords:** Anomiidae, Bivalvia, *Heteranomia*, *Isomonia*, jingle shell, *Pododesmus*, saddle oyster, synonyms

## Abstract

The family Anomiidae exhibits taxonomic ambiguity due to high morphological plasticity and overlapping diagnostic traits. In this study, we resurrect two anomiid species, *Heteranomia aculeata* and *Pododesmus glaucus*, and reclassify *Isonomia umbonata* as *Pododesmus umbonatus*. Our genetic analyses reveal interspecific *cox1* distances between *H. aculeata* and its former synonym *H. squamula* , *P. glaucus* and its synonym *P. squama*, and *P. umbonatus* relative to other *Pododesmus* species. Phylogenetic reconstructions based on five gene fragments (*cox1*, *16S rRNA*, *18S rRNA*, *28S rRNA*, and *histone H3*) corroborate the distinction among these previously synonymized species and confirm the placement of *P. umbonatus* in *Pododesmus*. Our morphological examinations further delineate diagnostic differences in adductor muscle scars compared to their congeneric species, reinforcing the resurrection and reclassification of these species. Under this reclassification, all Anomiidae genera are monophyletic, with *Pododesmus* as the earliest‐diverging lineage based on available data. However, Placunidae, which contains *Placuna* as the only genus, is recovered as sister to *Anomia*, rendering Anomiidae paraphyletic and indicating the need for a taxonomic revision of Placunidae. Additionally, we provide identification keys for the genera *Heteranomia* and *Pododesmus*. This study not only highlights the previously underestimated diversity of Anomiidae but also offers essential genetic resources and a refined taxonomic framework, advancing phylogenetic studies of Anomiidae and informing conservation strategies for morphologically complex marine taxa.

## Graphical abstract

The family Anomiidae displays taxonomic ambiguity due to morphological plasticity. We resurrect *Heteranomia aculeata* and *Pododesmus glaucus*, while reclassifying *Isonomia umbonata* as Pododesmus umbonatus. Phylogenetic analyses confirm these revisions and the monophyly of all genera within Anomiidae, with *Pododesmus* as the earliest‐diverging lineage. Additionally, we highlight the paraphyly of Anomiidae due to the recognition of Placunidae at the family level and provide identification keys for *Heteranomia* and *Pododesmus*, enhancing diversity understanding and informing conservation strategies for these understudied bivalves.

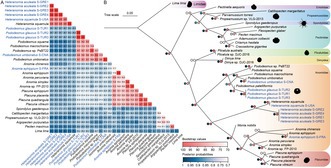

## 1 Introduction

Marine bivalves of the family Anomiidae Rafinesque, 1815 (order Pectinida Gray, 1854)—commonly known as jingle shells or saddle oysters—are distinguished from closed relatives like plicatulids and dimyids by their irregular, thin, flattened, and often translucent shells, which commonly attach to hard substrates via a calcified byssus emerging through a characteristic notch in the lower valve (Jeffreys 1863; Yonge 1977; Audino et al. 2024). The family includes nine extant genera (*Anomia* Linnaeus, 1758, *Enigmonia* Iredale, 1918, *Heteranomia* Winckworth, 1922, *Isomonia* Dautzenberg & Fischer, 1897, *Monia* Gray, 1850, *Patro* Gray, 1850, *Placunanomia* Broderip, 1832, *Pododesmus* Philippi, 1837, and *Tedinia* Gray, 1853) and three fossil genera (*Carolia* Cantraine, 1838, *Placunopsis* Morris & Lycett, 1853, and *Wakullina* Dall, 1896) (MolluscaBase 2025a). Anomiids exhibit global distribution, inhabiting environments ranging from intertidal zones to bathyal depths exceeding 1000 m (Huber 2010; Yonge 1977).

Taxonomic delineation within Anomiidae remains contentious, particularly at the genus level, due to overlapping morphological traits and limited molecular data (Huber 2010; Coan and Valentich‐Scott 2012; Albayrak and Çağlar 2024). Environmental factors, including substrate type and growth conditions, drive significant intraspecific shell variation, complicating species identification (Kolotukhina and Kulikova 2016; Moore 1984). Historical classifications relied heavily on variable shell characteristics—such as hinge structure, ornamentation, coloration, and muscle scar patterns—leading to synonymization issues. For instance, *Heteranomia squamula* (Linnaeus, 1758) and *Pododesmus squama* (Gmelin, 1791) have accumulated 20 and eight synonyms, respectively, reflecting early overinterpretation of shell variability (MolluscaBase 2025a). Previous studies have identified a range of additional informative morphological features for bivalves, such as shell microstructure, larval characteristics, and soft anatomical traits including musculature, alimentary systems, gill structures, and sperm ultrastructure (Yonge 1977; Bieler et al. 2014). However, these features have not been widely used in the taxonomy of Anomiidae, and the use of soft‐tissue characters for taxonomic classification is often constrained by the need for well‐preserved specimens and the limited availability of comprehensive data across species. Additionally, discrepancies in diagnostic features complicate taxonomy: Yonge (1977) proposed *H. squamula* as possessing a single rounded muscle scar, whereas Holmes (2017) documented specimens of this species with two oval scars. Similarly, *Isomonia umbonata* (Gould, 1861) has been inconsistently classified under *Placunanomia*, or as a subgenus of *Monia* or *Anomia* of Anomiidae due to conflicting interpretations of shell morphology (Huber 2010). Consequently, many historically recognized taxa have been synonymized or repeatedly revised due to morphological confusion and the lack of comprehensive molecular studies.

Advances in DNA barcoding, particularly using the mitochondrial cytochrome c oxidase subunit I (*cox1*) gene, have transformed molluscan taxonomy by providing objective criteria for species delimitation (Hubert and Hanner 2015; Lin et al. 2016; Papadopoulos et al. 2024). However, molecular studies on Anomiidae remain sparse. While some studies considered *P. squama* a synonym of *P. patelliformis* (Linnaeus, 1761), Holmes (2017), employing DNA barcoding and phylogenetic analyses, confirmed that the two species, both present in British waters, are distinct and that the joined muscle scar condition alone is not a reliable distinguishing characteristic of the two species. Besides, broader phylogenetic analyses primarily focused on the genus *Anomia* have suggested potential paraphyly within the family (Lin et al. 2024; Plazzi et al. 2011). Compounding these challenges, some sequences of *Anomia ephippium* Linnaeus, 1758 in public databases (e.g., GenBank accession numbers KX713191 for *16S rRNA*, KX713358 for *28S rRNA*, and KX713513 for *histone H3*) exhibit high similarities (99.39%–100%) with the sequences of *P. patelliformis* (Combosch et al. 2017), and the *cox1* sequence of *P. patelliformis* is identical to that of *Modiolus barbatus* (GenBank accession number MN064590) (Couton et al. 2019), suggesting the necessity of reexamination of these sequences.

Here, we obtained several specimens of Anomiidae that were collected from the Mediterranean and are morphologically identified as *H. aculeata* (Müller 1776) and *P. glaucus* (Monterosato, 1884), as well as *A. ephippium* , *H. squamula* , *I. umbonata*, *P. rudis* (Broderip, 1834), *P. patelliformis* , and *P. macrochisma* (Deshayes, 1839). These specimens had some dry tissues for DNA extraction, except *P. rudis* , *P. patelliformis, and* *P. macrochisma* , which were clean shells that could be used for morphological comparison only. Our preliminary analyses revealed that their *cox1* sequences differ by more than 5% from their putative conspecifics, *H. squamula* or *P. squama*, suggesting that *H. aculeata* and *P. glaucus* represent distinct evolutionary lineages that were erroneously synonymized. In addition, we found that *Isomonia umbonata* exhibits less sequence divergence with *Pododesmus* spp., suggesting that it may belong to *Pododesmus*. Our objectives are to (1) resurrect *H. aculeata* and *P. glaucus* as valid species; (2) reclassify *I. umbonata* into the genus *Pododesmus*; (3) clarify diagnostic characters for these species; and (4) determine the phylogenetic positions of these species. This work highlights the critical role of genetic distances and phylogenetics in uncovering hidden diversity and informing conservation in marine bivalves.

## 2 Materials and Methods

### 2.1 Sample Collection

Specimens of Anomiidae were collected from global localities by local fishermen to represent the target taxa. *Heteranomia aculeata* (*n* = 4, air‐dried tissues) and *P. glaucus* (*n* = 3, air‐dried tissues) were obtained from trawled rocks in the Saronic Gulf, Greece (90 m depth, September 2024) and Bozcaada Island, Turkey (100 m depth, August 2009), respectively. A single specimen of *H. squamula* with air‐dried tissues was collected from a lobster trap in Gloucester, Massachusetts, USA (50 m, September 2008). Fresh specimens of *A. ephippium* and *P. umbonatus* were sampled from the intertidal zone of Plage Chef de Baie, France (April 2016) and Dalian, China (20 m depth, April 2025), respectively. Empty shells of *P. rudis* (San Jose Gulf, Argentina, 15 m depth, September 2019), *P. patelliformis* (Saronic Gulf, Greece, 60 m depth, May 2022), and *P. macrochisma* (Port Hardy, British Columbia, Canada, 80 m depth, April 2008) were sourced from personal collections for morphological comparison.

### 2.2 Morphological Observations

Shell dimensions (length and height) were measured using a vernier caliper. Larger specimens were photographed with a Canon EOS 5D Mark IV camera (Japan), while muscle scars and smaller specimens were imaged using a Nikon MZ1270i Digital Stereo Microscope Imaging System (Japan). Voucher specimens were deposited in the Tropical Marine Biodiversity Collections of the South China Sea (SCSMBC), Chinese Academy of Sciences, Guangzhou, China.

### 2.3 DNA Extraction and Sequencing

Genomic DNA was extracted from air‐dried tissues of *A. ephippium* , *H. aculeata* , *H. squamula* , *P. glaucus* , and *P. umbonatus* using the CTAB method (Stewart and Via 1993). DNA quality and concentration were assessed via 1.0% agarose gel electrophoresis and NanoDrop ND‐1000 spectrophotometer (Thermo Scientific, USA), respectively. Five marker gene fragments, three nuclear (*18S rRNA*, *28S rRNA*, *histone H3*) and two mitochondrial (*cox1*, *16S rRNA*), were amplified using KOD One PCR Master Mix (Toyobo, Japan) per the manufacturer's protocol with published primers: LCO1490 and HCO2198 for *cox1* (Folmer et al. 1994), LRJ and 16SA for *16S rRNA* (Baco‐Taylor 2002; Ratnasingham and Hebert 2007), F19 and R1843 for *18S rRNA* (Elwood et al. 1985; Turbeville et al. 1994), D1R and LSUB for *28S rRNA* (Litaker et al. 2003; Scholin et al. 1994), and *H3F and H3R* for *histone H3* (Colgan et al. 1998). PCR products were purified and bidirectionally sequenced on an ABI PRISM 3730xl DNA Analyzer (Thermo Fisher Scientific, USA). Sequences were assembled using SeqMan (DNASTAR, USA).

### 2.4 Phylogenetic and Genetic Distance Analyses

Phylogenetic relationships were reconstructed from 31 Pectinida species and a Limidae Rafinesque, 1815 outgroup using the five marker genes, with the completeness of the dataset for each gene ranging from 71.43% to 76.19% (GenBank data; Table 1). Analyses were conducted using PhyloSuite v1.2.2 (Zhang et al. 2020) with multiple tools: (1) MAFFT v7.520 (Katoh and Standley 2013) for alignment under the “normal alignment” mode and the “auto” option; (2) Gblocks v0.91b (Talavera and Castresana 2007) for removing ambiguously aligned regions, with missing data or gaps filled with “‐”; (3) ModelFinder v1.5.4 (Kalyaanamoorthy et al. 2017) for selecting the best‐fit evolutionary model for each fragment based on the Bayesian Information Criterion under the partition mode; (4) Bayesian inference (BI) and maximum likelihood (ML) analyses were conducted using MrBayes v3.2.6 (Ronquist et al. 2012) and IQ‐TREE2 v2.1.2 (Nguyen et al. 2015), with the K3Pu + F + I + G4, K3Pu + F + I + G4, TIM2e + I + I + R2, TIM2e + I + I + R2, TIM2e + I + I + R models for the fragments of *cox1*, *16S rRNA*, *18S rRNA*, *28S rRNA*, and *histone H3*, for 10 million generations and 100,000 ultrafast bootstraps, respectively (Minh et al. 2013). Pairwise *cox1* genetic distances were calculated using the Kimura 2‐parameter (K2P) model in MEGA v12 (Kumar et al. 2024).

**TABLE 1:** Accession numbers of selected genes used for phylogenetic analyses.

Family	Genus	Species	*cox1*	*16S rRNA*	*18S rRNA*	*28S rRNA*	*histone H3*
Anomiidae	*Anomia*	*simplex*	KF850693	JN133626	—	—	—
		*ephippium*	KF369196	—	—	—	—
		*ephippium* S‐FRA^a^	PV769132	PV791198	PV770965	PV770955	PV785866
		*chinensis*	MN608245	—	—	AB105361	—
		sp. FP2010	GQ166573	GQ166557	—	—	—
		*peruviana*	KF850703	—	—	—	—
	*Pododesmus*	*umbonatus*	AB076951	—	—	AB102738	—
		*umbonatus* S‐CAN^a^	PV769131	PV791197	PV770961	PV770954	PV785865
		*glaucus* S‐TUR1^a^	PV769128	PV791194	PV770962	PV770951	PV785862
		*glaucus* S‐TUR2^a^	PV769129	PV791195	PV770963	PV770952	PV785863
		*glaucus* S‐TUR3^a^	PV769130	PV791196	PV770964	PV770953	PV7858644
		*macrochisma*	KF644022	—	AF229627	—	—
		*patelliformis*	—	KC429261	KC429342	KC429441	KC429179
		sp. PsBT22	OP347785	—	—	—	—
		*squama*	MG935056	—	—	—	—
	*Heteranomia*	*aculeata* S‐GRE1^a^	PV769123	PV791189	PV770956	PV770946	PV785857
		*aculeata* S‐GRE2^a^	PV769124	PV791190	PV770957	PV770947	PV785858
		*aculeata* S‐GRE3^a^	PV769125	PV791191	PV770958	PV770948	PV785859
		*aculeata* S‐GRE4^a^	PV769126	PV791192	PV770959	PV770949	PV785860
		*squamula* S‐USA^a^	PV769127	PV791193	PV770960	PV770950	PV785861
		*squamula*	OZ181722	OZ181722	—	—	—
	*Monia*	*nobilis*	—	—	MF077380	AJ307555	—
Placunidae	*Placuna*	*vitreum*	PP711111	PP599757	PP599752	PP599761	PP663639
		*ephippium*	PP711114	PP599760	PP599755	PP599765	PP663642
		*quadrangula*	PP711113	PP599759	PP599754	PP599764	PP663641
		*placenta*	KC429104	HQ840731	KC429343	KC429442	KC429180
Dimydae	*Dimya*	*lima*	—	KX713213	KC429344	KX713375	KC429181
		sp. DJC‐2016	—	—	KX713288	KX713376	KX713532
Plicatuloidea	*Plicatula*	sp. DJC‐2016	—	—	KX713337	KX713424	KX713573
		*australis*	—	—	AF229626	AB102737	KC429178
Entoliidae	*Pectinella*	*aequoris*	—	—	—	MH464049	MH464038
Spondylidae	*Spondylus*	*gaederopus*	JF496776	KR676345	KT757808	KT757854	KT757896
Propeamussiidae	*Catillopecten*	*margaritatus*	OQ434059	OQ434059	OQ427653	—	—
	*Parvamussium*	*torresi*	—	MH464019	MH464099	MH464043	MH464032
	*Propeamussium*	sp. VLG‐2013	KC429103	KC429259	KC429340	KC429437	KC429176
Pectinidae	*Argopecten*	*purpuratus*	KP265825	JN848518	EU660809	—	EU379526
	*Adamussium*	*colbecki*	—	HM600752	MH464058	FJ263652	EU379491
	*Chlamys*	*hastata*	—	FJ263648	MH464068	FJ263658	FJ263667
	*Crassadoma*	*gigantea*	—	EU379444	L49050	FJ263654	EU379498
	*Flexopecten*	*glaber*	HQ197900	MH490816	AJ389662	AJ307545	JQ611569
	*Pecten*	*maximus*	KC429102	X82501	L49053	KC429436	KC429175
Out group	*Lima*	*lima*	AF120649	KC429257	KC429339	AJ307558	JQ611555

*Note:* —: Data unavailable.

^a^ : Sequences produced by the study.

### 2.5 Divergence Time Estimation

The divergence time was estimated using MCMCtree as implemented in PAML v4.9h (Yang 2007), based on the maximum likelihood (ML) tree constructed previously. Fossil constraints were applied to relevant nodes based on data obtained from the Paleobiology Database (https://paleobiodb.org/). These included the emergence of Pteriomorphia Beurlen, 1944 (486.4–484.3 million years ago, MYA), and the earliest fossil records of Pectinidae Rafinesque, 1815 (387.9–382.3 MYA), Propeamussiidae Abbott, 1954 (241.5–237.0 MYA), and Anomiidae (323.4–306.0 MYA). The Markov chain Monte Carlo (MCMC) analysis was run for 10 million generations, with the first 1 million generations discarded as burn‐in. Parameters were sampled every 1000 generations, resulting in a total of 10,000 samples.

## 3 Results

### 3.1 Genetic Distances Prompt Species Reevaluation

**FIGURE 1:**
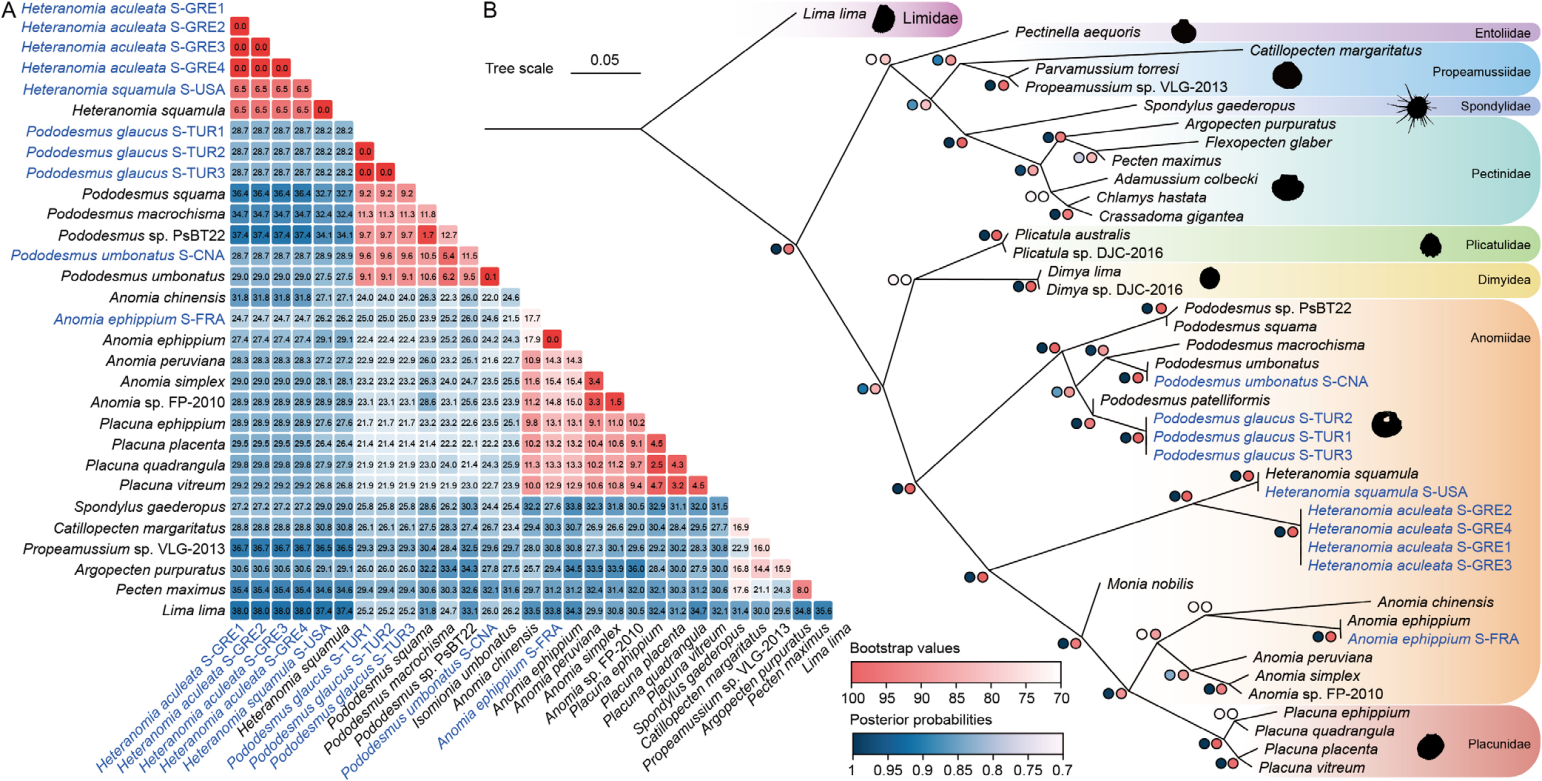
Genetic distance and phylogenetic relationships among the order Pectinida. (A) Kimura‐2‐parameter (K2P) genetic distances (%) based on *cox1*. (B) Phylogenetic placement of two resurrected species in Pectinida based on the maximum likelihood (ML) topology inferred from a 3,356‐bp concatenated alignment (*cox1*‐*16S rRNA*‐*18S rRNA*‐*28S rRNA*‐*histone H3*), with *Lima vulgaris* (Link, 1807) as the outgroup. Bootstrap values from ML analysis and posterior probabilities from Bayesian inference (BI) analysis are given at the nodes (ML/BI). The scale bar shows the number of substitutions per site. Sequences generated from this study are highlighted in blue. GenBank accession numbers for the gene sequences are provided in Table 1.

Sequencing of the target *cox1* fragments yielded 721–760 bp. After alignment and trimming, a 692 bp *cox1* matrix (21 sequences) was analyzed using the K2P model. Intraspecific K2P genetic distances for *H. aculeata* , *H. squamula* , *P. glaucus* , *P. umbonatus* , and *A. ephippium* ranged from 0% to 0.1%, indicating minimum intraspecific variations (Figure 1A). In contrast, interspecific distances revealed substantial divergences of the synonyms *H. squamula* and *H. aculeata* , exhibiting a K2P genetic distance of 6.5%. Similarly, the synonyms *P. glaucus* and *P. squama* showed a 9.2% K2P genetic distance, with 9.1%–11.3% divergences from other *Pododesmus* species. These results indicate that *H. aculeata* and *P. glaucus* should be reconsidered as distinct species. Additionally, *Placunanomia umbonata* Gould, 1861, currently recognized as *Isomonia umbonata*, is most closely related to species of *Pododesmus*, exhibiting 5.4%–11.3% K2P genetic distances from *Pododesmus* species, similar to the distances between other *Pododesmus* species. This result suggests that the generic status of *I. umbonata* should also be reevaluated.

### 3.2 Phylogenetic Analyses Reveal Anomioid Relationships

Alignment, trimming, and concatenation of *cox1* (692 bp), *16S rRNA* (700–765 bp), *18S rRNA* (1764–1766 bp), *28S rRNA* (1508–1525 bp), and *histone H3* (314–328 bp) sequences produced a 5708 bp matrix for 31 Pectinida species and a Limidae outgroup. The BI and ML phylogenetic trees exhibited mostly congruent topologies (Figure 1B), consistent with prior studies (Audino et al. 2020; Lin et al. 2024). Specimens of *H. aculeata* , *H. squamula* , *P. glaucus* , *P. umbonatus* , and *A. ephippium* each formed distinct, strongly supported clades (bootstrap = 100; posterior probability = 1.0), confirming they are distinct species (Figure 1B). The *H. aculeata* sequences form a sister clade to *H. squamula* . Similarly, *P. glaucus* formed a sister relationship with *P. patelliformis* , whereas *P. squama* occupied the most early‐diverging position within the *Pododesmus* lineage, validating the distinct status of *P. glaucus* . Besides, our results indicate that *Pododesmus* is paraphyletic, with *Isomonia umbonata* nested within this genus, forming a sister clade to *P. macrochisma* . Therefore, to preserve the generic monophyly of *Pododesmus*, we proposed to transfer *Isomonia umbonata* to *Pododesmus* and give the species a new name, *Pododesmus umbonatus*. Then, all Anomiidae genera are monophyletic based on available data, with *Pododesmus* as the earliest‐diverging lineage. However, the Placunidae Rafinesque, 1815 clade, a family with *Placuna* as the only genus, appeared a sister group to *Anomia* at the terminal branch of Anomiidae, rendering Anomiidae paraphyletic (Figure 1B).

**FIGURE 2:**
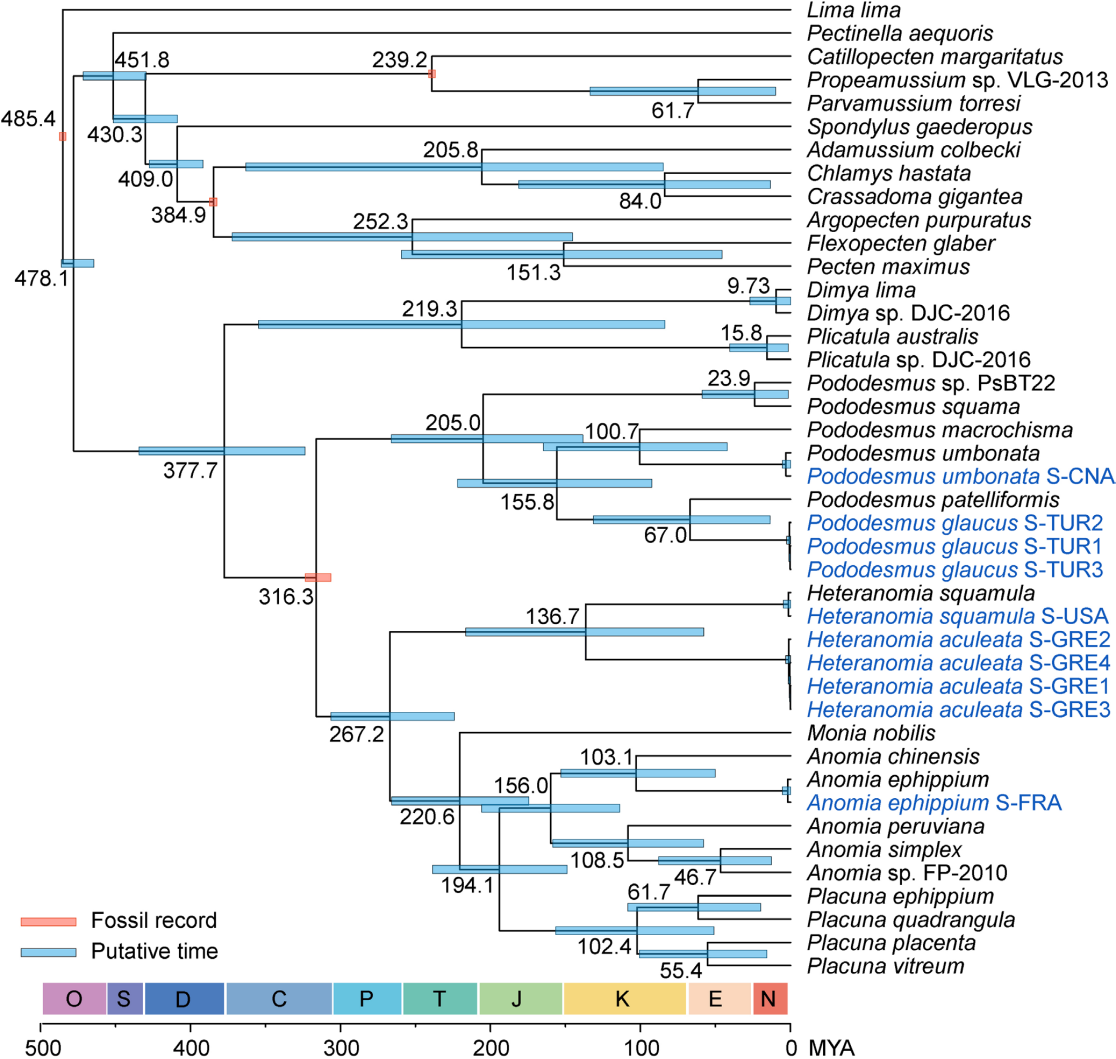
Divergent times of the order Pectinida. The topology was derived from Figure 1B, and the tree was calibrated at four nodes indicated by orange bars using fossil records. The putative time ranges are indicated by blue bars, and the length indicates the 95% confidence interval. The sequences produced in this study are labeled in blue.

Fossil‐based calibration of the tree indicates that the common ancestor of anomiids, dimyids, and plicatulids diverged around 377.7 MYA during the Upper Devonian (Figure 2). Consistent with the phylogenetic analyses above, the specimens of *P. glaucus* , *H. squamula* , and *H. aculeata* each exhibited very recent divergence times (Figure 2). In contrast, the divergence between the ancestors of *P. squama* and *P. glaucus* was estimated at approximately 205.0 MYA, while that between *H. squamula* and *H. aculeata* occurred around 136.7 MYA, further supporting the distinct evolutionary trajectories of these species.

## 4 Taxonomy

**Order:** PECTINIDA Gray, 1854

**Family:** ANOMIIDAE Rafinesque, 1815

*Heteranomia* Winckworth, 1922

Type species: *Heteranomia squamula* (Linnaeus, 1758)

**Diagnosis**

Shell convex, byssally cemented Anomiidae with two rounded muscle scars or one completely fused long‐oval scar; crurum straight. Rectum anterior retractor absent, subcrural groove sometimes pointed, supradorsal fusion of mantle lobes present. Outer ligament layers united, hypobranchial gland absent (Yonge 1977; Holmes 2017).

***Heteranomia aculeata* (Müller, 1776) (Resurrected Species)**

**Synonym:** *Anomia aculeata* Müller, 1776; *Monia aculeata* (Müller, 1776); *Monia aculeata var. laevis* Dautzenberg & Fischer, 1912

ZooBank: urn:lsid:zoobank.org:act:604A7262‐8272‐46CB‐976A‐9CDC0D642144

**Material Examined**

SCSMBC240261‐SCSMBC240264, Saronic Gulf, Greece, 90 m depth.

**Diagnosis**

*Heteranomia* with radially arranged, spine‐like projections extending from umbo to shell margin; inner surface with a single oval muscle scar fused from adductor and posterior byssal retractor scars; lower valve with a small, rounded adductor muscle scar beneath the byssal notch.

**Description**

**FIGURE 3:**
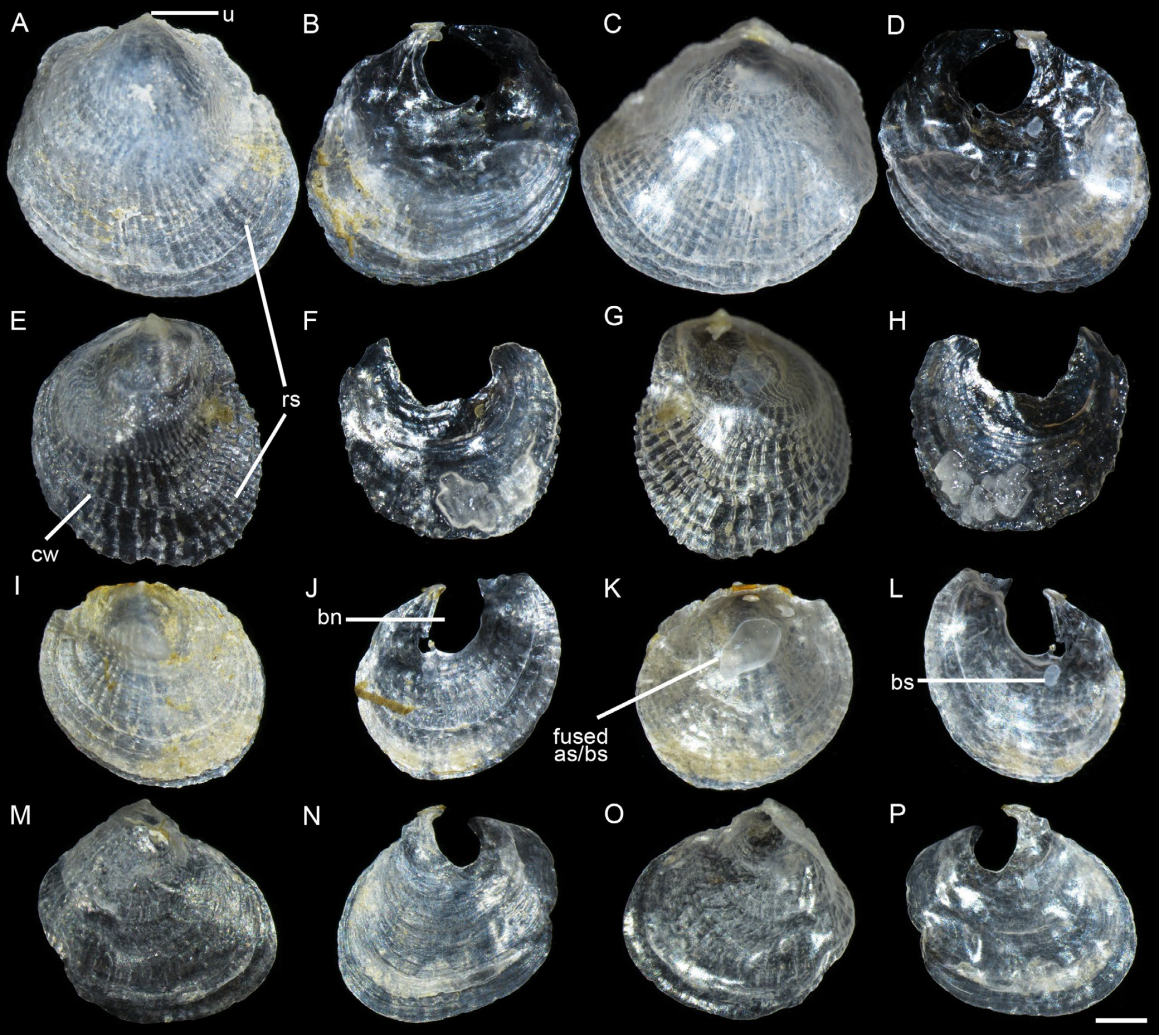
Shell morphology of *Heteranomia aculeata*. (A–D) SCSMBC240261, external (A, B) and internal (C, D) views of the upper and lower valves, respectively. (E–H) SCSMBC240262, external (E, F) and internal (G, H) views of the upper and lower valves, respectively. (I–L) SCSMBC240263, external (I, J) and internal (K, L) views of the upper and lower valves, respectively. (M–P) SCSMBC240264, external (M, N) and internal (O, P) views of the upper and lower valves, respectively. Scale: 1 mm. as, adductor muscle scar; bs, byssal retractor muscle scar; bn, byssal notch; cw, commarginal wrinkle; u, umbo; rs, radial spine.

Shell (Figure 3) up to 6 mm, nearly equidimensional (Table 2), oval to rounded, convex, substrate‐dependent, semitransparent. Upper valve outer surface with radial spines from the umbo, deviating along commarginal wrinkles; terminal and prominent umbones; inner surface smooth with a white, oval, completely fused adductor and byssal retractor scar. Lower valve thinner, fragile, transparent, with a prominent, rounded byssal notch and a white, rounded byssal retractor muscle scar.

**TABLE 2:** Shell morphology of the specimens based on upper valves.

Specimen	Length (mm)	Height (mm)	Ornamentation	Umbo	Color	Adductor muscle scar
*Heteranomia aculeata* GRE1	5.7	5.5	Radial spines	Terminal	White to colorlessness	Completely fused
*H. aculeata* GRE2	4.5	4.9	Radial spines	Terminal	White to colorlessness	Completely fused
*H. aculeata* GRE3	4.4	4.1	Radial spines	Terminal	White to colorlessness	Completely fused
*H. aculeata* GRE4	4.6	4.4	Radial spines	Terminal	White to colorlessness	Completely fused
*H. squamula*	9.4	10.0	Weakly commarginal wrinkles	Terminal	White to colorlessness	Two, adjoining
*Pododesmus glaucus* TUR1	42.9	43.9	Weakly commarginal wrinkles	Subterminal	Greenish to glaucous, orange‐yellow margin	Two, separated
*P. glaucus* TUR2	48.1	45.2	Weakly commarginal wrinkles	Subterminal	Greenish to glaucous, orange‐yellow margin	Two, separated
*P. glaucus* TUR3	42.1	41.5	Weakly commarginal wrinkles	Subterminal	Greenish to glaucous, orange‐yellow margin	Two, separated
*P. umbonatus*	14.5	15.1	Faintly radially striated	Terminal	Greenish to glaucous	Two, separated
*P. macrochisma*	66.7	68.7	Dense radial spines	Subterminal	External rufous, internal Greenish	Two, separated
*P. patelliformis*	51.2	46.6	Weakly commarginal wrinkles	Subterminal	White	Two, separated
*P. rudis*	28.7	28.9	Radial spines on margin	Terminal	Brown to yellow	Two, adjoining

**Distribution**

Mediterranean regions of Greece and North Atlantic regions of Denmark and Norway (Müller 1776).

**Remarks**

**FIGURE 4:**
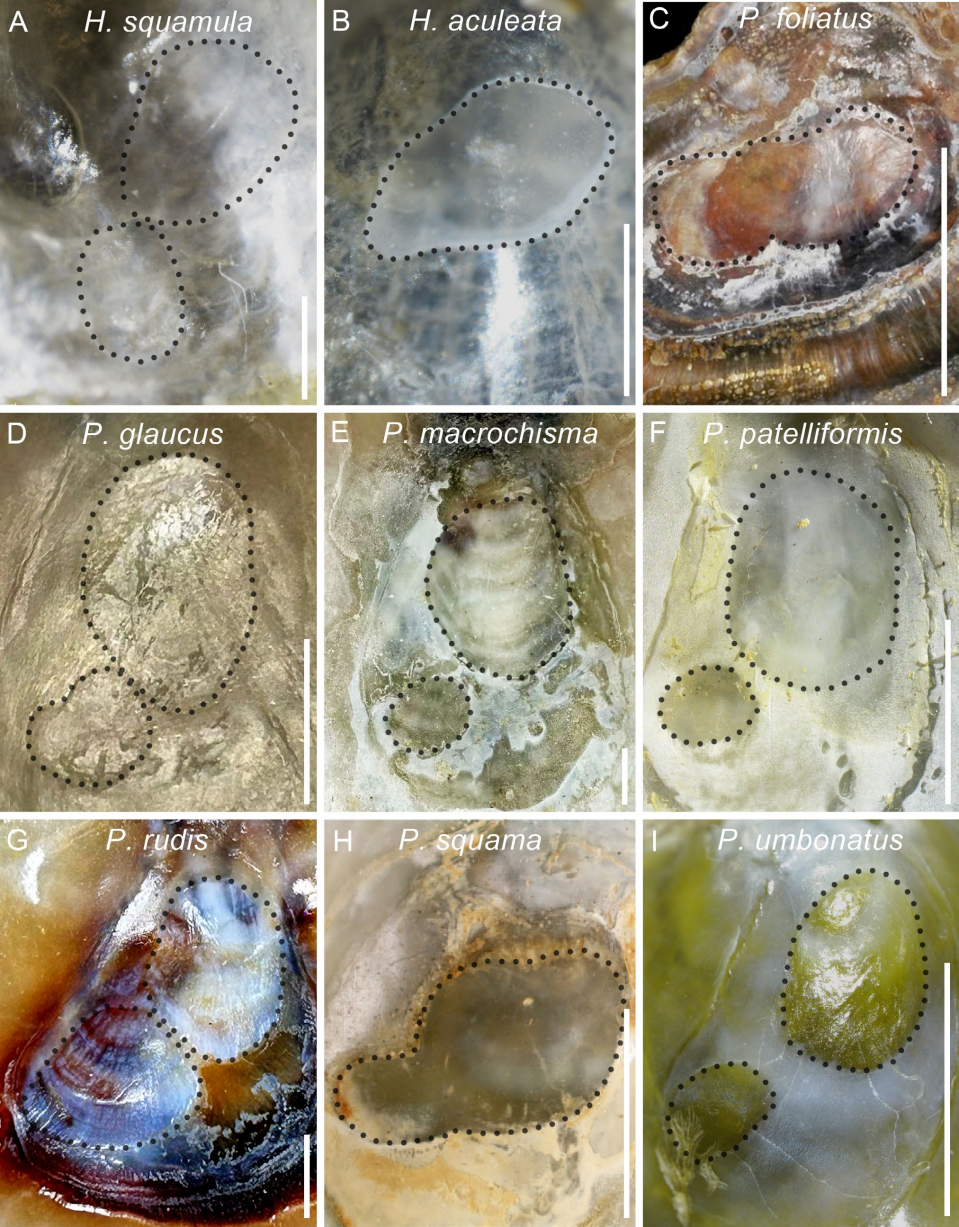
Photographs illustrating the muscle scars of *Heteranomia* and *Pododesmus*. (A) Separated muscle scars of *H. squamula* . (B) Completely fused muscle scars of *H. aculeata* . (C) Jointed muscle scars of *P. foliatus* (USNM715921, https://naturalhistory.si.edu/). (D–G) Separated muscle scars of four *Pododesmus* species. (H) Fused muscle scars of *P. squama* (Holmes 2017). (I) Separated muscle scars of *P. umbonatus* , SCSMBC240268. Scale: A–B, 1 mm; C–I, 5 mm.

Previous studies synonymized *Heteranomia aculeata* and *H. squamula* based on morphological comparisons, arguing that the two species are identical in having two muscle scars despite differences in the outer surface sculpture of their upper valves (Winckworth 1922; Merrill 1962; Peñas et al. 2006). However, this synonymization has been challenged since Yonge (1977) contested the original interpretation by asserting that *H. squamula* possesses only a single muscle scar in its upper valve. Our morphological examination resolves this ambiguity. Specimens of *H. aculeata* exhibit a distinct white, oval scar on the inner surface of the upper valve, formed by the complete fusion of the adductor and posterior byssal retractor muscle scars (Figures 3 and 4A,B). By contrast, *H. squamula* exhibits two separate scars. Complementing these morphological findings, our genetic analyses, including phylogenetic reconstructions, divergence time estimation, and interspecific distance metrics, reveal clear divergence between the two species (Figures 1 and 2).

*Pododesmus* Philippi, 1837

**Synonym:** *Anomia* (*Pododesmus*) Philippi, 1837; *Paranomia* Conrad, 1860; *Pododesmus* (*Pododesmus*) Philippi, 1837

Type species: *Pododesmus rudis* (Broderip, 1834)

**Diagnosis**

Byssally cemented Anomiidae with two rounded muscle scars; convex crurum; present rectum anterior retractor; pointed subcrural groove; no supradosal mantle lobe fusion; nonunited outer ligament layers; hypobranchial gland present; symmetrical anterior hemibranchs (Yonge 1977).

***Pododesmus glaucus* (Monterosato,** **1884** **) (Resurrected Species)**

**Synonym:** *Monia glauca* Monterosato, 1884

ZooBank:urn:lsid:zoobank.org:act:1AFD8F0E‐2A07‐4127‐B9AB‐50F6DA1879BD

**Material Examined**

SCSMBC240265–SCSMBC240267, Bozcaada Island, Turkey, 100 m depth.

**Diagnosis**

*Pododesmus* with upper valve sculptured by weakly commarginal wrinkles (irregular, concentric ridges representing pronounced, episodic pauses in shell growth) and orange radial streaks (orange bands extending from umbo to shell margin); greenish to glaucous umbones and shell center, orange‐yellow margin; unequal, irregularly oval, separated muscle scars; extremely fragile, semitransparent lower valve.

**Description**

**FIGURE 5:**
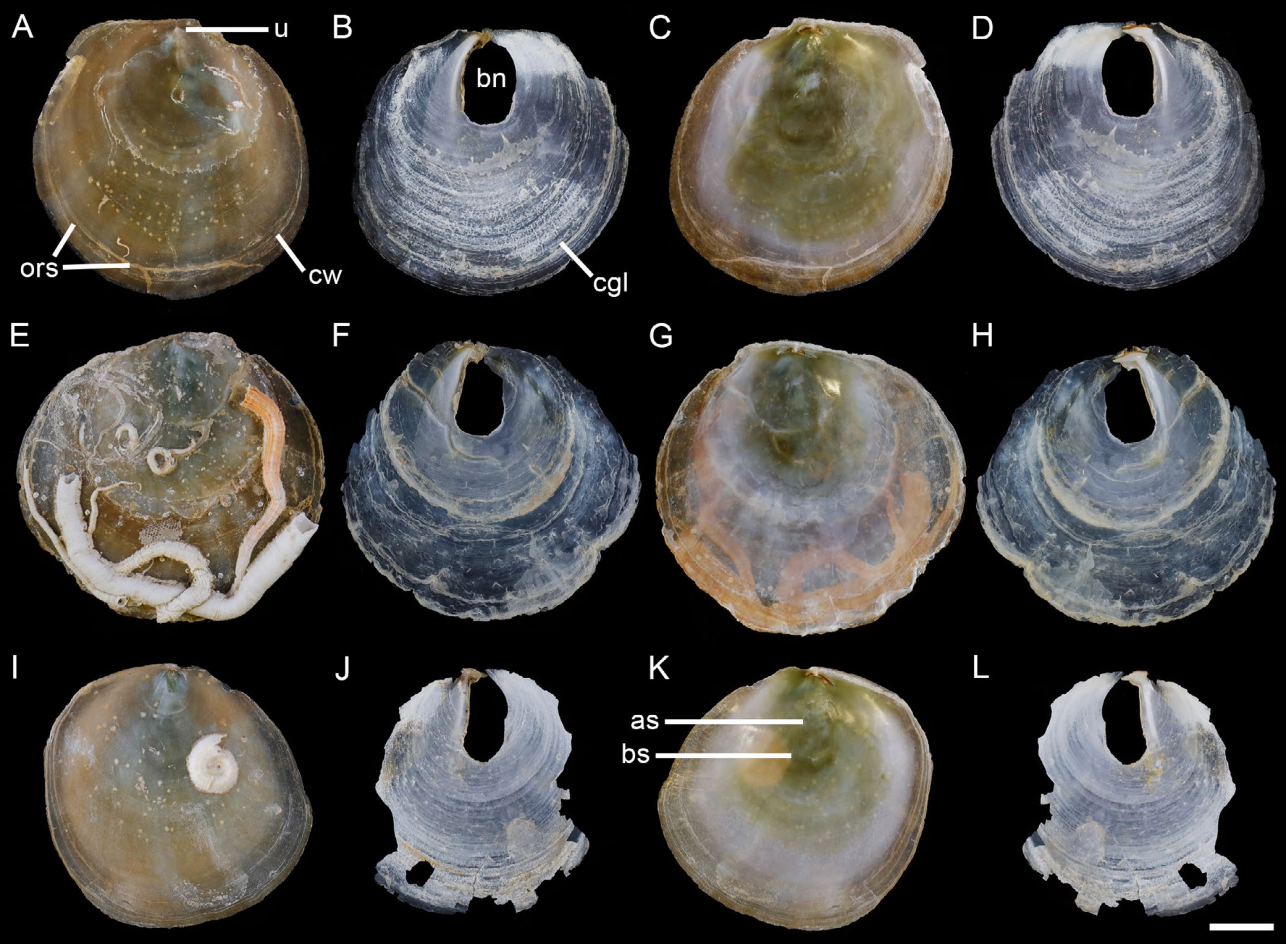
Shell morphology of *Pododesmus glaucus*. (A–D) SCSMBC240265, external (A, B) and internal (C, D) views of the upper and lower valves, respectively. (E–H) SCSMBC240266, external (E, F) and internal (G, H) views of the upper and lower valves, respectively. (I–L) SCSMBC240267, external (I, J) and internal (K, L) views of the upper and lower valves, respectively. Scale: 10 mm. as, adductor muscle scar; bs, byssal retractor muscle scar; bn, byssal notch; cgl, commarginal growth line; cw, commarginal wrinkle; ors, orange radial streak; u, umbo.

Shell (Figure 5) up to 44 mm, nearly equidimensional (Table 2), rounded, semitransparent. Upper valve with weak commarginal wrinkles and orange radial streaks from umbones; greenish to glaucous center, orange‐yellow margin; subterminal umbo; smooth inner surface, glaucous to white and orange‐yellow dorsoventrally; inconspicuous, unequal, oval, separated muscle scars. Lower valve fragile, semitransparent, with distinct commarginal growth lines (fine and concentric lines), a prominent oval byssal notch, and an inconspicuous byssal retractor muscle scar.

**Distribution**

Mediterranean regions of Italy, Greece, and Turkey (Monterosato 1884).

**Remarks**

*Pododesmus glaucus* was reported from Italy, Greece, and Turkey, while Winckworth (1922) synonymized this species with the widely distributed European *P. squama*. Huber (2010) supported this synonymization, arguing that Mediterranean specimens attributed to *P. glaucus* lacked sufficient morphological distinction from *P. squama*, particularly in features such as overlapping or closely positioned muscle scars, whitish to brownish radial streaks, and nearly smooth valves with faint irregular ridges. Although he also noted that Mediterranean specimens were typically smaller, rarely exceeding 25 mm in size, compared to *P. squama*. However, our findings challenge this synonymization: specimens matching Monterosato's (1884) original description of *P. glaucus* consistently exceed 40 mm in length (Table 2), contradicting Huber's size‐based argument. Crucially, we observed that the adductor and posterior byssal retractor muscle scars, though subtle, are fully separated in *P. glaucus* (Figure 3D). This feature distinguishes it not only from *P. squama* (Gmelin, 1791) but also from *P. foliatus* (Broderip, 1834) and *P. rudis* (Broderip, 1834). Furthermore, *P. glaucus* exhibits a unique glaucous upper valve with vivid orange radial streaks, a trait absent in congeners (Grant and Williams 2018). These morphological distinctions are corroborated by genetic and phylogenetic analyses (Figure 1), which reveal significant divergence between *P. glaucus* and related species. Combined, these analyses strongly support reinstating *P. glaucus* as a valid species within *Pododesmus*.

***Pododesmus* *u* *mbonatus* (Gould, 1861)**

**Synonym:** *Isonomia umbonata* (Gould, 1861); *Anomia lunula* Yokoyama, 1922; *Anomia pustulosa* Adams, 1861; *Anomia radulina* Adams, 1861; *Anomia sematana* Yokoyama, 1922; *Monia umbonata* (Gould, 1861); *Placunanomia radiata* Sowerby, 1914; *Placunanomia umbonata* Gould, 1861

ZooBank:urn:lsid:zoobank.org:act:EDDAF57F‐0E19‐475F‐9CF9‐C42785EFDBF3

**Material Examined**

SCSMBC240268, Dalian, Liaoning Province, China, about 20 m depth.

**Diagnosis**

*Pododesmus* with convex, smooth upper valve; faintly radially striated upper valve with terminal umbo; inner surface with widely separated scars; lower valve with acute‐ovate perforation.

**Distribution**

Sea of Japan to the South China Sea (Huber 2010; Zhang et al. 2016).

**Description**

**FIGURE 6:**
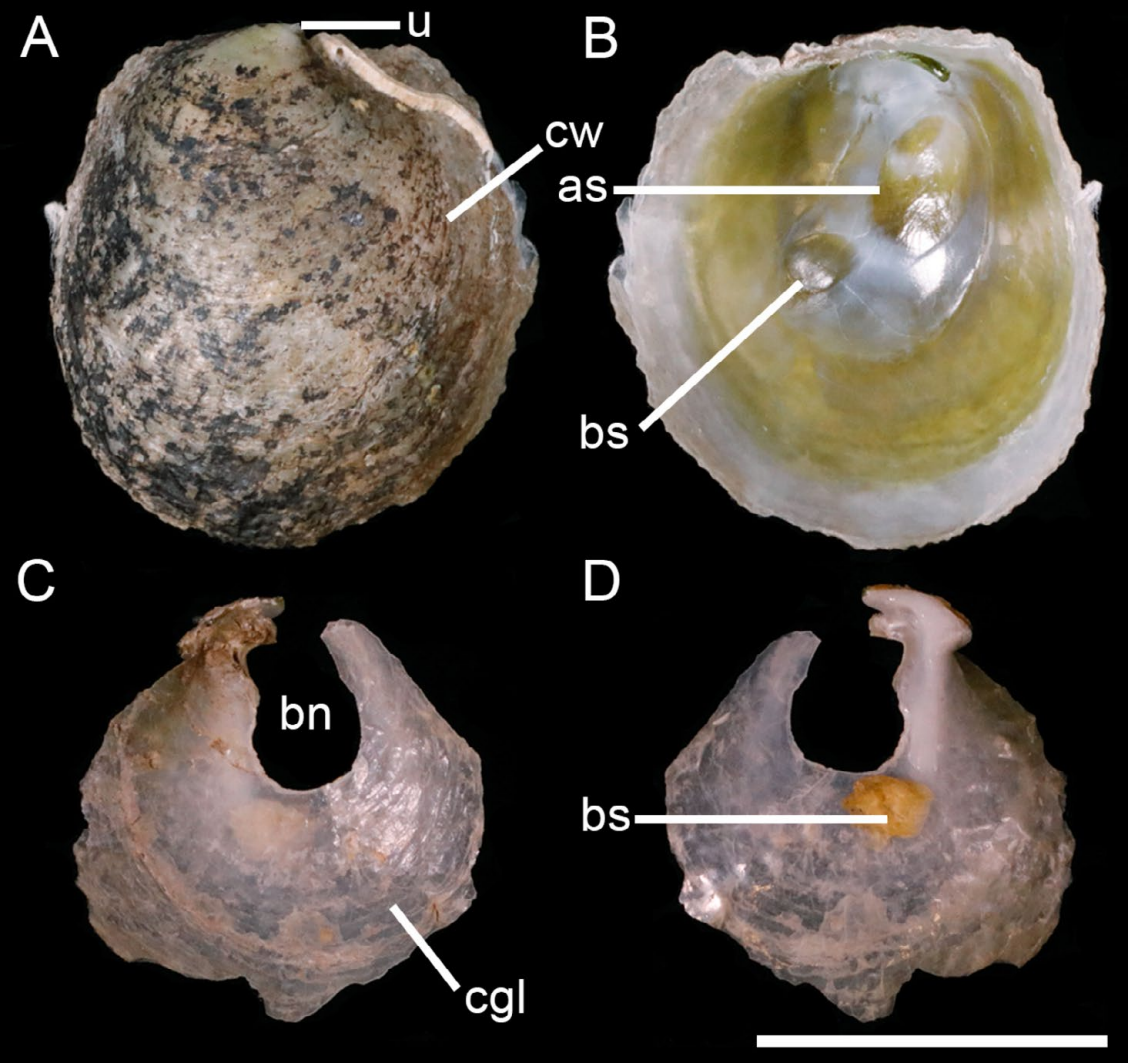
Shell morphology of *Pododesmus umbonatus*. (A–D) SCSMBC240268, external (A–C) and internal (B–D) views of the upper and lower valves, respectively. Scale: 10 mm. as, adductor muscle scar; bs, byssal retractor muscle scar; bn, byssal notch; cgl, commarginal growth line; cw, commarginal wrinkle; u, umbo.

Shell (Figure 6) about 15 mm, slightly higher than long (Table 2), oval, semitransparent. Upper valve external surface silver to light green, with weak commarginal wrinkles, terminal umbones; smooth inner surface, greenish to glaucous, margin white; clear, unequal, oval, separated muscle scars. Lower valve fragile, semitransparent, with weakly commarginal growth lines, a prominent oval byssal notch, and a byssal retractor muscle scar.

**Remarks**

Previous morphological studies distinguished *Pododesmus umbonatus* from *Pododesmus* based on its fragile, rather flat, and almost equilateral pectiniform shell in adults, as well as its deeper‐water habitat (approximately 200 m, Huber 2010). However, our discovery of *P. umbonatus* in the shallow waters of China's Bohai Sea (intertidal to 20 m depth) demonstrates that this species has a broader bathymetric range than previously recognized. Huber (2010) further questioned its placement in *Pododesmus*, arguing that only the two muscle scars resemble those of the genus and suggesting it might represent an undescribed species. In contrast, our phylogenetic analyses strongly support its classification within *Pododesmus* (Figure 1), corresponding to the phylogenetic results of Holmes (2017) based on *cox1* fragments. Morphologically, *P. umbonatus* is readily distinguishable from congeners such as *P. foliatus* , *P. rudis* , and *P. squama* by its separated adductor and posterior byssal retractor muscle scars (Figure 4). Additional diagnostic traits include its convex shell and wide distance between these scars, which further differentiate it from other *Pododesmus* species, including the resurrected *P. glaucus* .

## 5 Discussion

Early taxonomic descriptions, constrained by limited specimens and technology, often relied on morphological traits of specimens from a particular locality, leading to frequent revisions (Braby et al. 2024). This resulted in numerous synonyms, for example, *Mytilus edulis* Linnaeus, 1758, with 22 synonyms, and repeated reclassifications of genera like *Pecten* Müller, 1776 (MolluscaBase 2025b, 2025c), complicating species identification and diversity assessments (Dubois 2008; Honey and Scoble 2001). Modern molecular techniques, such as DNA barcoding, have provided robust tools to resolve taxonomic ambiguities in groups with high morphological plasticity (Papadopoulos et al. 2024; Tan et al. 2022). Re‐evaluating controversial taxa with molecular tools is essential to validate synonymies, refine classifications, and preserve taxonomic accuracy (Dias et al. 2018; Weis et al. 2014). Our morphological and phylogenetic analyses resurrected *H. aculeata* and *P. glaucus* as valid species, revealing underestimated diversity within the morphologically variable family Anomiidae. Our results highlight the need to reassess many synonymized taxa, as historical classifications based solely on morphology may obscure valid species due to limited sampling or technological constraints (Schutze et al. 2015).

The taxonomic history of *P. umbonatus* reflects persistent confusion, with assignments to *Placunanomia*, *Monia*, and *Isomonia* and five synonymized species due to the morphological variability (Huber 2010). A prior *cox1* sequence for *P. umbonatus* was reported without comparison to other Anomiidae (Matsumoto 2003), while phylogenetic analyses placed this sequence into the clade with *P. patelliformis* and *P. squama* (Holmes 2017), indicating the need for taxonomic revision. Our phylogenetic analyses place *P. umbonatus* within *Pododesmus*, being a sister species to *P. macrochisma* (Figure 1B). Besides, its morphological features, especially the two widely separated muscle scars (Figure 4), also support this reclassification. The sole remaining *Isomonia* species, *I. alberti* (Dautzenberg and Fischer 1897), lacks recent records, images, or molecular data and exhibits confluent scars similar to *P. foliatus* and *P. squama* (Dautzenberg and Fischer 1897; Holmes 2017; Huber 2010). To synonymize *Isomonia*, we would need to examine the material of *I. alberti* , but the absence of data for this species necessitates retaining it as a valid genus pending further study. Morphological and molecular analyses of *I. alberti* are critical for resolving Anomiidae taxonomy.

Molecular techniques enhance species identification, while substantial sequence discrepancies can complicate taxonomic interpretations. Our sequence alignments revealed notable differences between our *A. ephippium* sequences and some previously published data (Combosch et al. 2017). While the *cox1* sequence from Combosch et al. (2017) shows a high identity (99.85%) with our data, we observed considerable divergences in other markers: *16S rRNA* (78.17%), *28S rRNA* (90.89%), and *histone H3* (87.90%). These divergent sequences show 99.39%–100% identity with the corresponding genes of *P. patelliformis* and, given that Combosch et al. (2017) sequenced both species concurrently, potential cross‐contamination could be one plausible explanation. This possible contamination may lead to the misidentification of paraphyletic *Anomia* in prior studies (Lin et al. 2024, 2025). By excluding these sequences, we recovered a monophyletic *Anomia* clade, leading to a refined understanding of anomiid relationships based on available data. Besides, there are three variants of *A. ephippium 18S rRNA* sequences, including (1) KX713269 and AF120535 from the western Mediterranean by Combosch et al. (2017) and Giribet and Wheeler (2002), respectively, (2) AJ389661 from the northern Mediterranean by Steiner and Hammer (2000), and (3) PV770965 from the eastern Atlantic by this study, suggesting possible underlying biological complexities that warrant further investigation. Similarly, the problematic *P. patelliformis* *cox1* sequences and misclassifications in scallop studies (e.g., *Delectopecten* Stewart, 1930 misidentified as *Cyclopecten* Verrill, 1897) highlight the need for rigorous data validation to ensure accurate phylogenetic inference and species identification (Audino et al. 2020; Lin et al. 2023, 2025).

Our phylogenetic results align with prior studies but revealed *Anomia*'s monophyly, contradicting earlier findings based on incorrect data (Lin et al. 2024). We found that the genera of the superfamily Anomioidea are monophyletic based on available data, including *Placuna* Lightfoot 1786 from Placunidae. However, Placunidae is a sister clade to *Anomia* and renders Anomiidae paraphyletic, misrepresenting its evolutionary history. Historical classifications placed *Placunanomia* and *Placuna* in Placunidae based on byssal notch closure and ligament specialization, with *Placunanomia* similar to *Pododesmus* and *Placuna* resembling the more highly evolved *Anomia* (Huber 2010; Yonge 1977). However, *Placunanomia* is now reassigned to Anomiidae (Bolton and Portell 2013; MolluscaBase 2025a). Therefore, we hypothesize that *Placuna* represents a lineage diverging toward a free‐living lifestyle (Yonge 1977), and propose not to recognize Placunidae in future revision work, with the reclassification of *Placuna* directly into Anomiidae, or the resurrection of *Placuninae* Rafinesque, 1815 in Anomiidae. This adjustment would streamline Anomioidea into a monophyletic group, better reflecting bivalve evolutionary relationships. Unfortunately, limited specimen availability in this study precludes detailed morphological comparisons. Broader sampling, morphological analyses, and phylogenetic studies of Anomioidea are needed to elucidate its internal relationships and evolutionary history (Cárdenas et al. 2013).

**Key to the Species of *Heteranomia* Winckworth**, **1922**

1. Upper valve with radial spines and a muscle scar……*H.* *aculeata* (Müller, 1776);
  Upper valve smooth with two separate muscle scars……*H.* *squamula* (Linnaeus, 1758)

**Key to the species of *Pododesmus* Philippi, 1837**

1. Upper valve with merged muscle scars……2;
  Upper valve with separate muscle scars……4
2. Muscle scars unequal in size……*P. squama* (Gmelin, 1971);
  Muscle scars subequal in size……3
3. Merged scars merged with distinct boundary…*P.* *foliatus* (Broderip, 1834);
  Merged scars merged without distinct boundary…… *P. rudis* (Broderip, 1834)
4. Muscle scars widely separated…… *P. umbonatus* (Gould, 1861);
  Muscle scars close to each other……5
5. Upper valve semitransparent, glaucous with orange streaks……*P.* *glaucus* (Monterosata, 1884);
  Shell thick and opaque with falcate mantle scar……*P.* *macrochisma* (Deshayes, 1839);
  Upper valve sculptured by weak radial riblets……*P* *.* *patelliformis* (Linnaeus, 1761)

## 6 Conclusion

This study applied genetic distances, phylogenetics, and morphological analyses to resolve longstanding ambiguities in Anomiidae, resurrecting *H. aculeata* and *P. glaucus* as valid species and reclassifying *P. umbonatus* within *Pododesmus*. Our analyses provide robust evidence for these taxonomic revisions, revealing underestimated biodiversity within this understudied bivalve family. The monophyly of Anomiidae genera is confirmed, but the paraphyly of Anomiidae due to the recognition of Placunidae as a family suggests a need to revise Anomioidea's classification, potentially by integrating *Placuna* into Anomiidae. Our results advanced Anomiidae phylogenetics and informed conservation strategies for morphologically complex marine taxa by providing identification keys and a refined taxonomic framework. Future studies should prioritize broader sampling and molecular analyses of rare taxa, such as *I. alberti* , to further elucidate anomiid diversity and evolutionary relationships.

## Author Contributions

**Yi‐Tao Lin:** data curation (lead), visualization (lead), writing – original draft (lead). **Jian‐Wen Qiu:** conceptualization (lead), funding acquisition (lead), supervision (lead), writing – review and editing (lead).

## Conflicts of Interest

The authors declare no conflicts of interest.

## Data Availability

The gene sequences generated in this study were deposited in GenBank with accession numbers shown in Table 1.
